# 

**Published:** 1905-01-14

**Authors:** 


### NOTICE TO CORRESPONDENTS.

All MSS., Letters, Books for Review, and other matters intended for the Editor should be addressed The Editor, "The Hospital," The Hospital Buildings, 28 & 29 Southampton Street, Strand, W.C.

The Editor cannot undertake to return rejected MSS., even when accompanied by stamped directed envelope.

**Notice to Advertisers and Subscribers.**

All Advertisements, Orders for Copies of The Hospital, and Business Communications should be addressed to **THE MANAGER (Not to the Editor),** The Hospital, 28 & 29 Southampton Street, Strand, London, W.C.

The Cost of Subscription, post free, is as follows:—Per week, 3½d.; per quarter, 3s. 3d.; per half-year, 6s. 6d.; per annum, 13s. Foreign and Colonial:—Per annum, 19s. 6d., payable, in all cases, in advance.

**NOTICE.—Vol. XXXVI. of "The Hospital" (April to September 1904), in handsome cloth binding, is now on sale at the office of this Journal, price 10s. 6d. Cases for binding the Numbers contained in this Volume 2s. Index to Vol. XXXVI., 3½d., post free.**

**The Monthly part for January is now ready. Price 1s., by post, 1s. 3d.**

### BOOKS RECEIVED.

Kegan Paul, Trench, Trübner, and Co.

"Fever Nursing." By R. W. Wilcox, M.A.

Swan Sonnenschein and Co., Ltd.

"Premature Burial." By Tebb and Vollum. Second edition.

Edward Arnold.
"Human Embryology and Morphology." By A. Keith, M.D., F.R.C.S.

A. and C. Black.
"Cairo of To-day."

Baillière, Tindall and Cox.

"A Manual of Toxicology." By A. H. Brundage, M.D.

### MEDICAL APPOINTMENTS.

Edward M. Corner, M.A., M.B., F.R.C.S., Consulting Surgeon to the Bowes Park Cottage Hospital, vice the late Mr. H. W. Allingham.

**"The Hospital" Institutional Library.**

"Hospitals and Asylums of the World." 4 Vols., with a Portfolio of Plans, complete £12 12s.

"Burdett's Hospitals and Charities, 1904." 5s. net.

"Burdett's Official Nursing Directory." 8s. net.

"Cottage Hospitals, General, Fever, and Convalescent." 10s. 6d.

"Uniform System of Accounts for Hospitals and Public Institutions." New Edition. 4s. net.

"Hospital Expenditure: The Commissariat." 2s. 6d.

"A Legal Handbook for the Use of Hospital Authorities." 2s. 6d.

"The Cottage Hospital Case Book or Register of Patients." 10s. and 12s. 6d.

All these are published by the Scientific Press, Ltd., and may be obtained through any bookseller, or direct from the publishers, 28 and 29 Southampton Street, Strand, London, W.C.

### Medical Appointments.

DISTRICT INFIRMARY AND CHILDREN'S HOSPITAL, ASHTON-UNDER-LYNE.

WANTED, a RESIDENT HOUSE-SURGEON, with a double medical and surgical qualification and registered.

Salary £120 per annum, with Board, Washing, and Lodging, Gas, Firing, and Attendance. Previous experience of hospital work required.

The applicant should be able to give lessons to probationers. The number of beds occupied is about sixty on an average. Personal canvassing will disqualify. Duties to commence on March 15th, 1905.

Applications, enclosing copies of testimonials and stating age, to be sent by letter before JANUARY 21st, 1905, to the Honorary Secretary.

LEONARD BOTTOMLEY.

120 Stamford Street, Ashton-under-Lyne. (150)

### The Nurses' Bookshelf.

**THE NURSING PROFESSION: How & Where to Train.**

By Sir HENRY BURDETT, K.C.B.

2/- net; 2 4 post free.

**A HANDBOOK FOR NURSES.**

By J. K. WATSON, M.D.

5/- net; 5/4 post free.

**NURSING: ITS THEORY AND PRACTICE.**

By PERCY G. LEWIS, M.D.

3/6 post free.

**OPHTHALMIC NURSING.**

By Dr. STEPHENSON

3/5 net; 3/10 post free.

**ELEMENTARY ANATOMY AND SURGERY FOR NURSES.**

By McADAM ECCLES, M.D.

2/6 post free.

**ELEMENTARY PHYSIOLOGY FOR NURSES.**

By Dr. MARSHALL.

2/- post free.

**THE NURSES' PRONOUNCING DICTIONARY.**

2/- post free.

**PRACTICAL GUIDE TO SURGICAL BANDAGING AND DRESSINGS.**
By WM. JOHNSON SMITH. 2/- po t free.

**EXAMINATION OF THE URINE.**
By Dr. WATSON. 1/- net; 1/1 post free.

**NURSING NOTES ON MIDWIFERY AND GYNÆCOLOGY.**
By SISTER ROSS. 2/- net; 2/2 post free.

**A COMPLETE HANDBOOK OF MIDWIFERY.**

By Dr. WATSON.

6/- net; 6/4 post free.

**ART OF MASSAGE.**

By CREIGHTON HALE.

6/- post free.

**SURGICAL WARDWORK AND NURSING.**

By Dr. MILES.

3/6 net; 3/10 post free.

**THE SCIENTIFIC PRESS, LTD.**

*Publishers of Nursing Text-Books, Charts, and Case-Books of all Descriptions.*

SEND FOR NEW CATALOGUE.

**28 & 29 Southampton St., Strand, London, W.C.**

